# Development of a recombinase polymerase amplification assay for rapid detection of *Francisella noatunensis* subsp. *orientalis*

**DOI:** 10.1371/journal.pone.0192979

**Published:** 2018-02-14

**Authors:** Khalid Shahin, Jose Gustavo Ramirez-Paredes, Graham Harold, Benjamin Lopez-Jimena, Alexandra Adams, Manfred Weidmann

**Affiliations:** 1 Institute of Aquaculture, Faculty of Natural Sciences, University of Stirling, Stirling, Scotland, United Kingdom; 2 Aquatic Animal Diseases Lab, Division of Aquaculture, National Institute of Oceanography and Fisheries, Suez, Egypt; Oklahoma State University, UNITED STATES

## Abstract

*Francisella noatunensis* subsp. *orientalis* (*Fno*) is the causative agent of piscine francisellosis in warm water fish including tilapia. The disease induces chronic granulomatous inflammation with high morbidity and can result in high mortality. Early and accurate detection of *Fno* is crucial to set appropriate outbreak control measures in tilapia farms. Laboratory detection of *Fno* mainly depends on bacterial culture and molecular techniques. Recombinase polymerase amplification (RPA) is a novel isothermal technology that has been widely used for the molecular diagnosis of various infectious diseases. In this study, a recombinase polymerase amplification (RPA) assay for rapid detection of *Fno* was developed and validated. The RPA reaction was performed at a constant temperature of 42°C for 20 min. The RPA assay was performed using a quantitative plasmid standard containing a unique *Fno* gene sequence. Validation of the assay was performed not only by using DNA from *Fno*, closely related *Francisella* species and other common bacterial pathogens in tilapia farms, but also by screening 78 Nile tilapia and 5 water samples. All results were compared with those obtained by previously established real-time qPCR. The developed RPA showed high specificity in detection of *Fno* with no cross-detection of either the closely related *Francisella* spp. or the other tested bacteria. The *Fno-*RPA performance was highly comparable to the published qPCR with detection limits at 15 and 11 DNA molecules detected, respectively. The RPA gave quicker results in approximately 6 min in contrast to the qPCR that needed about 90 min to reach the same detection limit, taking only 2.7–3 min to determine *Fno* in clinical samples. Moreover, RPA was more tolerant to reaction inhibitors than qPCR when tested with field samples. The fast reaction, simplicity, cost-effectiveness, sensitivity and specificity make the RPA an attractive diagnostic tool that will contribute to controlling the infection through prompt on-site detection of *Fno*.

## Introduction

Francisellosis is an emergent systemic bacterial disease affecting the global production of tilapia. It is caused by *Francisella noatunensis* subsp. *orientalis* (*Fno*), which is a Gram negative facultative intracellular pathogen. The disease has been described in various geographical regions in warm water fish species including tilapia *Oreochromis* spp. [1–6], hybrid striped bass, *Morone chrysops* x *M*. *saxatilis* [7], three-line grunt *Parapristipoma trilineatum* [8] and ornamental fish [9, 10]. *Fno* is closely related to *Francisella noatunensis* subsp. *noatunensis* (*Fnn*) that affects commercially important cold-water fish including Atlantic cod, *Gadus morhua* L. [11, 12] and Atlantic salmon, *Salmo salar* L. [13, 14] and *F*. *philomiragia* which is an aquatic opportunistic bacterium that infects immunosuppressed mammals including humans [15–19]. Multiple-organ granuloma with high morbidity and variable mortalities are the main characteristics of Francisellosis in fish [20].

Diagnosis of *Fno* is a challenging issue due to its nature as a fastidious intracellular bacterium and the insufficient availability of sensitive and specific detection methods for this pathogenic aquatic microorganism [21]. Conventional diagnosis of *Fno* via bacterial isolation in culture media has many constrains as it takes several days to grow and is often overgrown by concomitant bacteria [22, 23]. Furthermore, affected tissue samples need homogenisation for maximum bacterial recovery and isolation by culturing can give false negative results [24]. Antibody-based immunological assays such as enzyme-linked immunosorbent assay (ELISA) and immunohistochemistry (IHC), have also been used for *Fno* diagnosis, but they were reported to have low sensitivity and limited throughput [25, 26].

Nucleic acid-based methods have been applied for *Fno* detection, including conventional polymerase chain reaction (PCR) [4, 5, 27–29], quantitative real time PCR (qPCR) [5, 21, 22, 30, 31], duplex PCR, *in situ* hybridisation [32] and loop mediated isothermal amplification (LAMP) [33]. Despite the fact that, these techniques have their own points of interest, downsides, for instance, time consuming [22, 33], labour intensive, prerequisite for skilled staff, liability to give false negative or false positive results due to low sensitivity or specificity [21], high influence with reaction inhibitors [34, 35] and requirement of complex design [33] make them more challenging to use for pond-site diagnosis.

The isothermal amplification technology recombinase polymerase amplification (RPA) is an alternative molecular technique that has been successfully used for field diagnosis of various pathogens. The technique has been widely used recently due to its affordable price (~4.5 USD per test), high sensitivity (limits of detection as low as 1 genome copy), short reaction time (results can be obtained in less than 10 min), robustness and simplicity (minimum equipment and hands-on manipulation required) [36] and has been used in a suitcase laboratory [37].

Since its first introduction in 2006, it has been widely adopted for the detection of pathogens of clinical importance in human medicine [38–45], veterinary medicine [46–53] and agriculture [54, 55]. In the aquatic veterinary field, the RPA has recently been developed for viral diseases for various fish and shell fish hosts including *Penaeus stylirostris* denso virus [56], shrimp white spot syndrome virus [57], infectious hypodermal and hematopoietic necrosis virus [58], Cyprinid Herpes virus-3 [59], abalone herpes-like virus and red-spotted grouper nervous necrosis virus [60]. There are no reported RPA assays developed for bacterial diseases affecting aquaculture until now.

The aim of the current study was to develop and validate a real-time RPA for a rapid and specific detection of *Fno* to be applied as a point-of-care diagnostic tool for monitoring and preventing the spread of francisellosis in tilapia aquaculture.

## Materials and methods

### Bacterial isolates and DNA extraction

In this study isolates of *Fno* and other bacteria including, closely related *Francisella* species and other non-related bacteria, were used for testing the specificity of the RPA. Bacterial isolates used are listed in Table 1. All *Francisella* strains including *Francisella noatunensis* subsp. *orientalis* (*Fno*), *F*. *noatunensis* subsp. *noatunensis* (*Fnn*) and *F*. *philomiragia* (*Fp*) were cultured from stock cultures on cysteine heart agar with 2% bovine haemoglobin (CHAH; BD, Oxford, UK). The agar plates were incubated at 28°C for 3 days for *Fno*, 22°C for 5 days for *Fnn* and 28°C for 24 h for *Fp* isolates respectively. After incubation, growth and purity confirmation, a loop-full of bacteria from each plate was inoculated into modified Mueller Hinton broth (MMHB) with 2% isovitalex and 0.1% glucose (BD, Difco, USA) and incubated in a shaking incubator (Kühner, Switzerland) at 28°C at 150 rpm for 20 h. Colonies of *Aeromonas hydrophila*, *Streptococcus agalactiae*, *S*. *iniae*, *Escherchia coli*, *Yersinia ruckeri* and *Pseudomonas* sp. were grown on Tryptic Soy agar (Sigma-Aldrich, UK) at 28°C for 48 h at 150 rpm, then inoculated into tryptic soy broth (Sigma-Aldrich, UK) and incubated for 24 h at 28°C at 150 rpm. Strains of *Vibrio anguillarum* and *Photobacterium damselae* were cultured on marine agar at 28°C for 48 hrs (Difco, USA) then inoculated into tryptic soy broth (Sigma-Aldrich, UK) with 2% NaCl (Sigma-Aldrich, UK) and incubated for 24 h at 28°C at 150 rpm.

**Table 1:** Bacterial strains tested in the study.

Bacterial species	Strain ID	Source
*F*. *noatunensis* subsp. orientalis*	STIR-GUS F2f7	Tilapia (UK)
*F*. *noatunensis* subsp. *orientalis*^*#*^	NVI-PQ1104	Tilapia (Costa Rica)
*F*. *noatunensis* subsp. *orientalis* ^*#*^	DSMZ21254^**T**^	Three-line grunt (Japan)
*F*. *noatunensis* subsp. *orientalis*^‡^	NVI-9449	Malawi cichlids (Austria)
*F*. *noatunensis* subsp. orientalis*	AVU-Fran-Cos1	Tilapia (Mexico)
*F*. *noatunensis* subsp. *orientalis*^*#*^	AVU-STIR-HON1	Tilapia (Central America)
*F*. *noatunensis* subsp. *noatunensis*^*#*^	NCIMB 14265 ^**T**^	Atlantic, Cod (Norway)
*F*. *noatunensis* subsp. *noatunensis* ^*#*^	NVI-7601	Atlantic Cod (Ireland)
*F*. *noatunensis* subsp. *noatunensis*^*#*^	PQ1106	Atlantic Salmon (Chile)
*F*. *philomiragia*^*#*^	ATCC^®^ 25015^**T**^	Muskrat (USA)
*F*. *philomiragia*^*#*^	ATCC^®^ 25017^**T**^	Water (USA)
*F*. *philomiragia*^*#*^	CCUG 12603^**T**^	Human abscess (Sweden)
*Aeromonas hydrophila*^§^	ATCC^®^ 7966^**T**^	Milk with fish odour (USA)
*Streptococcus agalactiae*^§^	ATCC^®^ 51487^**T**^	Tilapia (Israel)
*Streptococcus iniae*^§^	ATCC^®^ 29178^**T**^	Amazon fresh water dolphin
*Vibrio anguillarum*^§^	ATCC^®^ 19264^**T**^	Atlantic Cod (UK)
*Photobacterium damselae*^§^	ATCC^®^ 51736 ^**T**^	Yellow tail fish (Japan)
Escherichia coli*	ATCC^®^ 11775^**T**^	Urine (Sweden)
*Yersinia ruckeri*^§^	ATCC^®^ 29473^**T**^	Rainbow trout (USA)
Pseudomonas species*	AVU-STIR-Ps17	Lump sucker (UK)

^(T)^ Type strains

AVU: Aquatic Vaccine Unit bacterial culture collection, DSMZ: The German Collection of Microorganisms and Cell Cultures, NVI: The Norwegian Veterinary Institute, NCIMB: The National Collection of Industrial Food and Marine Bacteria, ATCC: American Type Culture Collection.

(*strains provided by aquatic vaccine unit, Stirling University

^§^ bacterial strains kindly provided by Dr. Kim Thompson, Aquatic Research Group, Moredun Research institute, UK

^*#*^ bacterial strain kindly donated by Dr. Duncan Colquhoun, Norwegian Veterinary Institute

^‡^ bacterial strain kindly provided by Professor El-Matbouli, University of Veterinary Medicine, Austria)

The genomic DNA from the different bacterial cultures was extracted using a real pure genomic DNA extraction kit (Real Laboratory, Valencia, Spain) following the manufacturer’s protocol for genomic DNA extraction from bacterial cells. The concentration of the DNA samples was measured using a nanodrop (Nanodrop 1000, ThermoFisher Scientific, UK). Each DNA sample was standardised to 100 ng/μL and stored at -20°C until use.

### Field samples

Samples of spleen (*n =* 78), head kidney (*n =* 78) and water (*n =* 5) were used in the current study. The tissue samples were obtained from 78 moribund and clinically healthy Nile tilapia, *Oreochromis niloticus* (L.) that were randomly collected from two different geographical locations including 38 fish from three tilapia farms in the UK (Farm one (Lincolnshire): 10 fish / 40±3 gm; Farm two (Lincolnshire): 10 fish /45±2 gm; Farm three (London): 18 fish / 12±5 gm) and 40 fish from a commercial tilapia farm in Prachinburi province, Thailand (10±2gm). The first and second UK farms and the Thai farm had a history of natural outbreaks of francisellosis during 2011–2012 and 2008, 2013–2014 respectively with granulomatous lesions in the affected fish and variable mortalities [4,29,33]. The third UK farm had no history of francisellosis, but it supplied red tilapia fry to an aquaponics farm in London, UK, where a natural outbreak of francisellosis occurred during spring 2017. Five water samples of 500 mL were collected from different sections at the infected aquaponics farm that received fish from the third UK farm including 1 sample from UV filter unit, 2 samples from bio-filter tanks and 2 samples from 2 separate tanks holding diseased fish.

Isolation of *Fno* from spleen samples from the first and second UK farms and the Thai farm was attempted using CHAH following the recommended protocol [28, 29]. DNA from 20 mg of the collected spleen and head kidney samples was extracted using the same kit used for bacterial gDNA extraction following the manufacturer’s instruction for tissues. DNA from 350 mL of each water sample was extracted using DNeasy Blood and Tissue Kit (QIAGEN, Germany) as described previously [61]. All the extracted DNA samples were standardised to 100 ng/μL and stored at -20°C until use.

### Preparation of plasmid DNA standard containing the FSC771 gene

#### Plasmid DNA cloning

A specific gene sequence unique to *Fno* [24] representing the FSC771 hypothetical protein gene (Genbank accession no. JQ780323.1) was synthesized and ligated into vector backbone pENTR221 (Geneart, Life Technologies Ltd, Paisely, United Kingdom). The resulting standard *Fno-*plasmid “pFNO STD”, (S1 Fig in supporting files) was transformed into an *E*. *coli* vector (OmniMAX™ 2 T1R) and purified from transformed bacteria using QIAprep8 Miniprep Kit (QIAGEN, UK). The final construct was verified by sequencing and the sequence congruence with in the insertion sites was 100%.

#### Restriction, concentration and quantification of the plasmid standard

The plasmid was linearized with Eco-RV (R01955 NEB, New England laboratories, UK), and the restricted plasmid band was extracted from a 1% agarose gel using a sterile scalpel and purified using the QIAEX^®^ II Gel extraction kit (QIAGEN, UK) following the manufacturer`s protocol. The concentration of the purified plasmid DNA was quantified using the Quant-iT™ PicoGreen® dsDNA kit (Molecular Probes, ThermoScientific, UK) following the kit protocol. The fluorescence (excitation = 480 nm and emission = 520 nm) was measured by plate reader (BioTek, Synergy HT, USA). Using the molecular weight of DNA, the copy numbers of the plasmid was determined and a quantitative plasmid standard ranging from 10^7^ to 10^1^ DNA molecules/**μ**L was prepared.

### Real-time qPCR for the plasmid standard

Real-time qPCR was performed according to a previously established protocol [22] using the primers listed in Table 2. The assay was performed on a LightCycler^®^ 2.0 (ROCHE, Germany) in a 20 **μ**L reaction volume that consisted of 0.3 **μ**M from each primer (Eurofins Genomics, UK), 1x Luminaris color HiGreen™ qPCR master mix (ThermoFisher Scientific, UK), 1 **μ**L DNA template and up to 20 **μ**L of nuclease free water (ThermoFisher Scientific, UK). The qPCR cycling conditions were adopted from the published qPCR [22] as following, 50°C for 2 min for uracil-DNA glycosylase enzyme activation, 95°C for 10 min to start denaturing the UNG enzyme and activate the DNA polymerase enzyme followed by 45 cycles at 95°C for 15 sec and 60°C for 1 min. Melting curve analysis formed of 1 cycle at 95°C for 30 sec, 55°C for 30 sec and 95°C for 30 sec. All samples were run in duplicates and each run included non-template control (Milli-Q water only). A standard curve was created from the data of three runs (*n =* 3).

**Table 2:** List of primers and probes used in the experiment.

Name	Sequence (5’– 3’)	Ampliconsize	Source
RPA (F1)	ATGAGATATGTGTTAATTTGGCTGTTCCTGTACGA	153 bP	This study
RPA (R2)	TAGTTGTATCAGTAATAGGCGTAACTCCTTTTAGC		
RPA (P)	GTATAATCTTTTCGTTCTAACTGAGATTGAXTXFTTCTAGGAAGCTAA-**PH**		
qPCR (F)	CATGGGAAACAAATTCAAAAGGA	85 bP	[22]
qPCR (R)	GGAGAGATTTCTTTTTTAGAGGAGCT		
PCR (F5)	CCTTTTTGAGTTTCGCTCC	1140 bP	[62]
PCR (F11)	TACCAGTTGGAAACGACTG		

(F) Forward primers, (R) Reverse primer, (P) probe, (PH) Phosphate group to block elongation.

### Real-time RPA primers and probe

**Fig 1:**
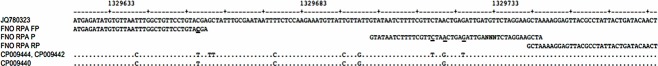
Alignment of RPA target region in unique *Fno* FSC771 hypothetical gene region. *Fno* sequences at the top, followed by primer sequences and *F*. *philomiragia* sequences at the bottom. The *Fno* target region is 100% conserved and present in JQ780323 (shown), CP006875, CP011921-23, CP012153, CP018051, NC_023029, LTDO01000001, CP003402, NC_017909. Positions are given in relation to *Fno* sequence CP006875. The nucleotide underlined at the 3’-end of forward primer (FNO RPA FP) is mismatched to *F*. *philomiragia* sequences following the ARMS concept. NNN represents the tetrahydrofuran bridge of the probe.

Two primers and an exo-probe for RPA were designed following the manufacturer instructions [63] using the *Fno* FSC771 hypothetical protein gene sequence (456 pb; GenBank accession no. JQ780323.1). All existing similar sequences in fish pathogenic *Fno*, *Fnn*, as well as the human pathogenic and environmental *Fp* strains [64–67] were included in the alignment to exclude cross detection (Fig 1).

The probe contained a tetrahydrofuran spacer (THF) with a 5’ quencher (BHQ1-dT; thymidine nucleotide carrying Blackhole quencher) and 3’ fluorescence reporter (FAM-dT; thymidine nucleotide carrying 6 carboxy-flourescein). The sequences of the final primers and probe used in this study are listed in Table 2. The primers and the probe for RPA were synthesised by TIB Molbiol (Berlin, Germany).

### RPA reaction

The RPA reaction was performed in a 50 μL volume using a TwistAmp™ exo lyophilized kit (TwistDX, Cambridge, United Kingdom). The reaction mixture included 420 nM of each primer, 120 nM FAM-tagged RPA probe, 14 mM magnesium acetate, 1x rehydration buffer and 1 μl of template. All the reagents except the template and Mg acetate were prepared in a master mix which was distributed into 0.2 mL tubes (Eppendorf, Germany). Four μL of Mg acetate was pipetted into the lid of the reaction tubes containing the dried reaction-pellet. One μL of the template was added to the mixture aliquots and quickly centrifuged using a mini-spin centrifuge (MCF 2360, LMS Co., Ltd., South Korea) then transferred to the reaction tubes and lids were closed carefully. The tubes were vigorously mixed by inversion 10 times and centrifuged for 20 sec. The tubes were immediately placed in an ESE Quant Tube Scanner device (QIAGEN, Germany). The tubes were incubated at 42°C for 20 min where the fluorescence measurement including excitation at 470 nm and detection at 520 nm for FAM channel was performed. After 4 min, the tubes were taken out of the ESE Quant Tube Scanner device for a quick spin then returned to complete the scanning. The ESE Quant scanner software enabled threshold validation including evaluation of fluorescence by increasing the fluorescence above three standard deviations over the background detected in the first minute of the reaction. In addition, the curve slope represented in mV/time can be utilized (slope adaptable) and a second derivative window for calculation of the turning point of the upward fluorescence development can be used for verifying curves with a very low slope.

### Analytical sensitivity and specificity of RPA detection

The quantitative *Fno*-plasmid DNA standard was used to evaluate the sensitivity of the RPA and qPCR reactions using 1 μL of a dilution range of 10^7^ to 10^1^ molecules/μL. Both RPA and qPCR were repeated 10 times using individual master mixes. Each run included duplicate reactions and non-template control (Milli-Q water only). To evaluate the specificity of the RPA reaction, the assay was tested using 1 μL of gDNA (100 ng/μL) from the different bacterial strains listed in Table 1.

### Clinical validation of *Fno* RPA

DNA extracted from fish tissues (78 spleen and 78 head kidney samples) and water samples (*n* = 5) were used to test the developed RPA. The tissue and water samples were firstly tested by both conventional PCR using previously published *Francisella* genus-specific primers [62] (Table 2) and qPCR using *Fno*-specific primers [22] targeting a region slightly downstream from the region used for RPA amplicon design. One **μ**L from the total DNA (100 ng/**μ**L) was amplified using RPA and results were compared with data obtained from qPCR. All positive RPA results were additionally verified by secondary derivative analysis as implemented in the analysis software. Samples tested negative by qPCR and positive by RPA were further diluted to 1:10 and 1:100 dilutions and re-tested to investigate potential inhibition of qPCR. The diagnostic performance of the developed RPA was evaluated by calculation of sensitivity, specificity, positive predictive value (PPV) and negative predictive value (NPV) using free statistical calculators “Diagnostic Test Evaluation Calculator” (https://www.medcalc.org/calc/diagnostic_test.php) [68] and results were presented as a percentage.

### Statistics

Microsoft Excel 2016 was used to arrange the data for analysis. GraphPad® prism v.6 (GraphPad, San Diego, CA, USA) was used to calculate a semi-log regression of the data set of 10 runs of *Fno-*RPA and qPCR by blotting the threshold time in minutes (Tt) for RPA or the cycle threshold (Ct) for qPCR against the molecules detected of the *Fno-*plasmid standard DNA dilutions (10^7^:10^1^ copies/**μ**L). A probit regression analysis was performed by Minitab^®^ v.17 (Minitab Ltd., UK) to calculate limits of detection (LOD) in 95% of the cases following both assays.

## Results and discussion

Francisellosis is one of the most serious bacterial diseases affecting the tilapia industry worldwide. Mortality rates of up to 95% were documented in cultured tilapia in Taiwan [69, 70] and more recently mortalities up to 40% in broodstock in Mexico [5] and 5–50% in fingerlings and juveniles in Brazil [31]. Application of rapid, sensitive and robust monitoring represents the most reliable strategy for early identification of outbreaks and initiation of control measures to prevent the spread of the disease.

**Fig 2:**
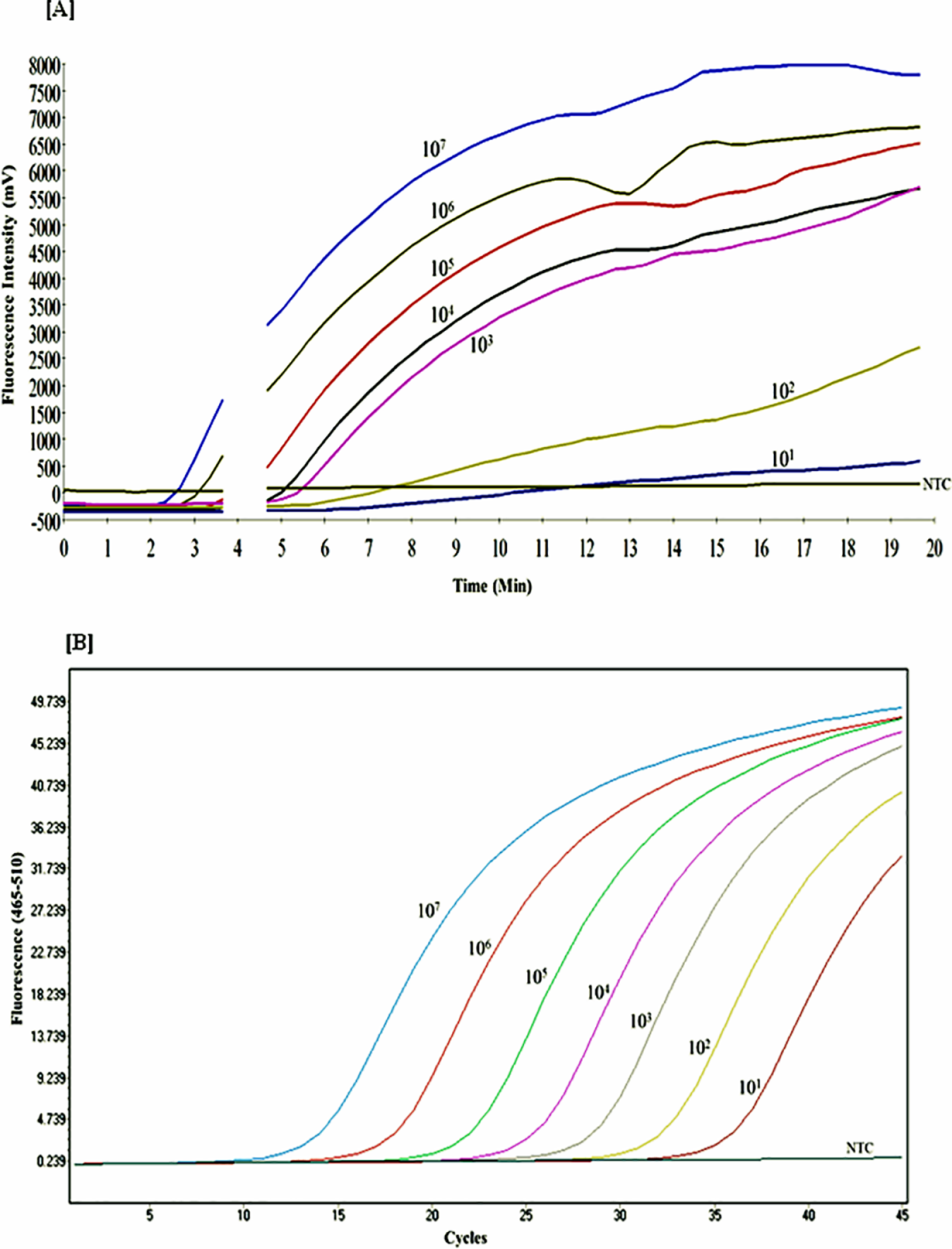
Performance of RPA and qPCR using dilutions of *Fno*-plasmid DNA standard. Representatives amplification curves from three runs of both RPA (A) and qPCR (B) (*n =* 3) showing the fluorescence development over time in both assays using a dilution range of 10^7^ to 10^1^ copies /**μ**L of the *Fno*-plasmid standard DNA. After 4 minutes, the tubes were mixed and centrifuged, therefore, a gap appears in the graph.

**Fig 3:**
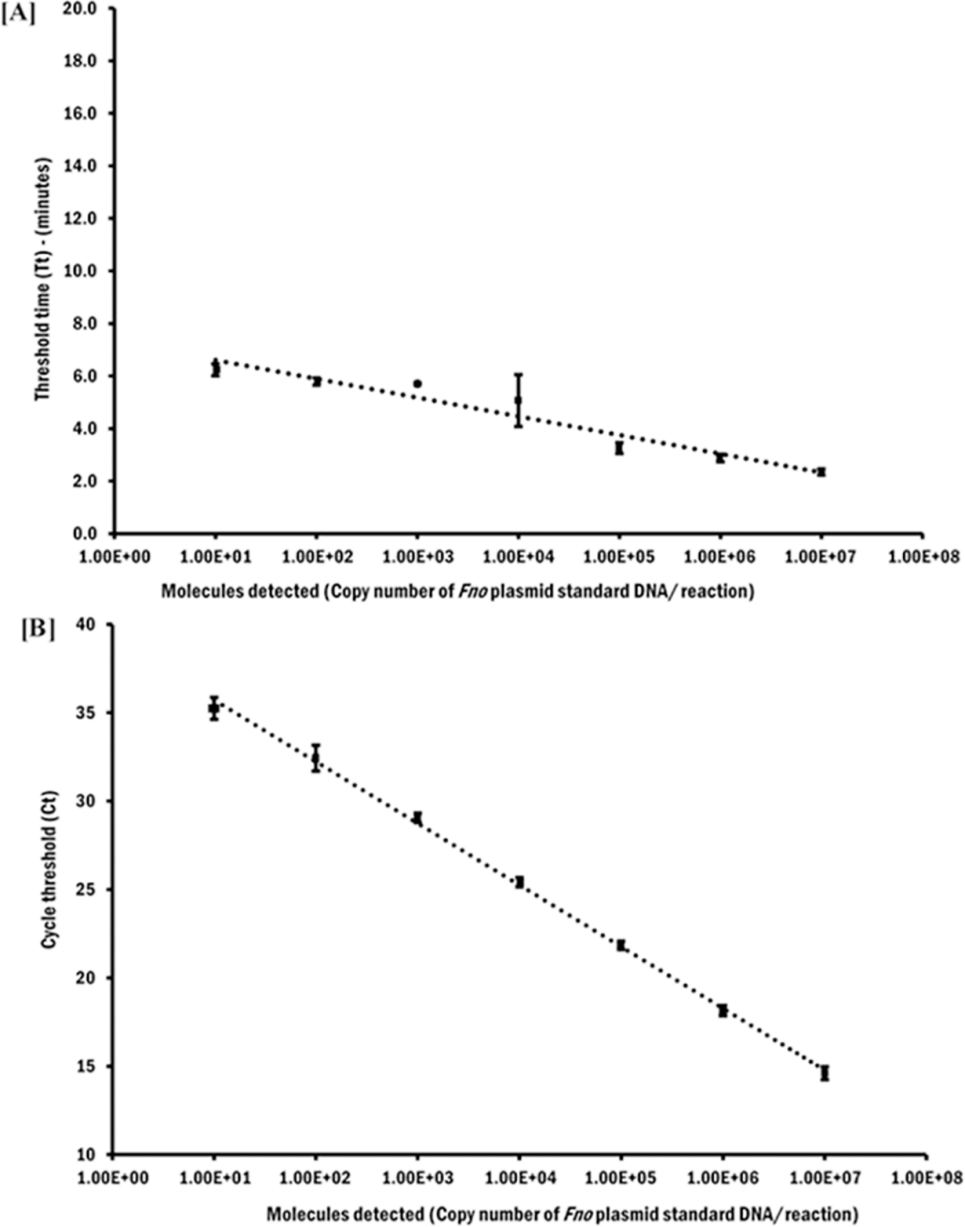
Reproducibility of RPA and qPCR assays. The semi-log regression generated by 10 data sets of RPA (A) and qPCR (B) on a dilution range of the molecular standard (10^7^−10^1^ DNA molecules/reaction).Threshold time (Tt in RPA) and cycle threshold (Ct in qPCR) were represented as a mean ± standard deviation (±SD). The highest detection sensitivity of both assay were 10 DNA molecules detected. These data were used for the probit regression analysis showed in Fig 4.

**Fig 4:**
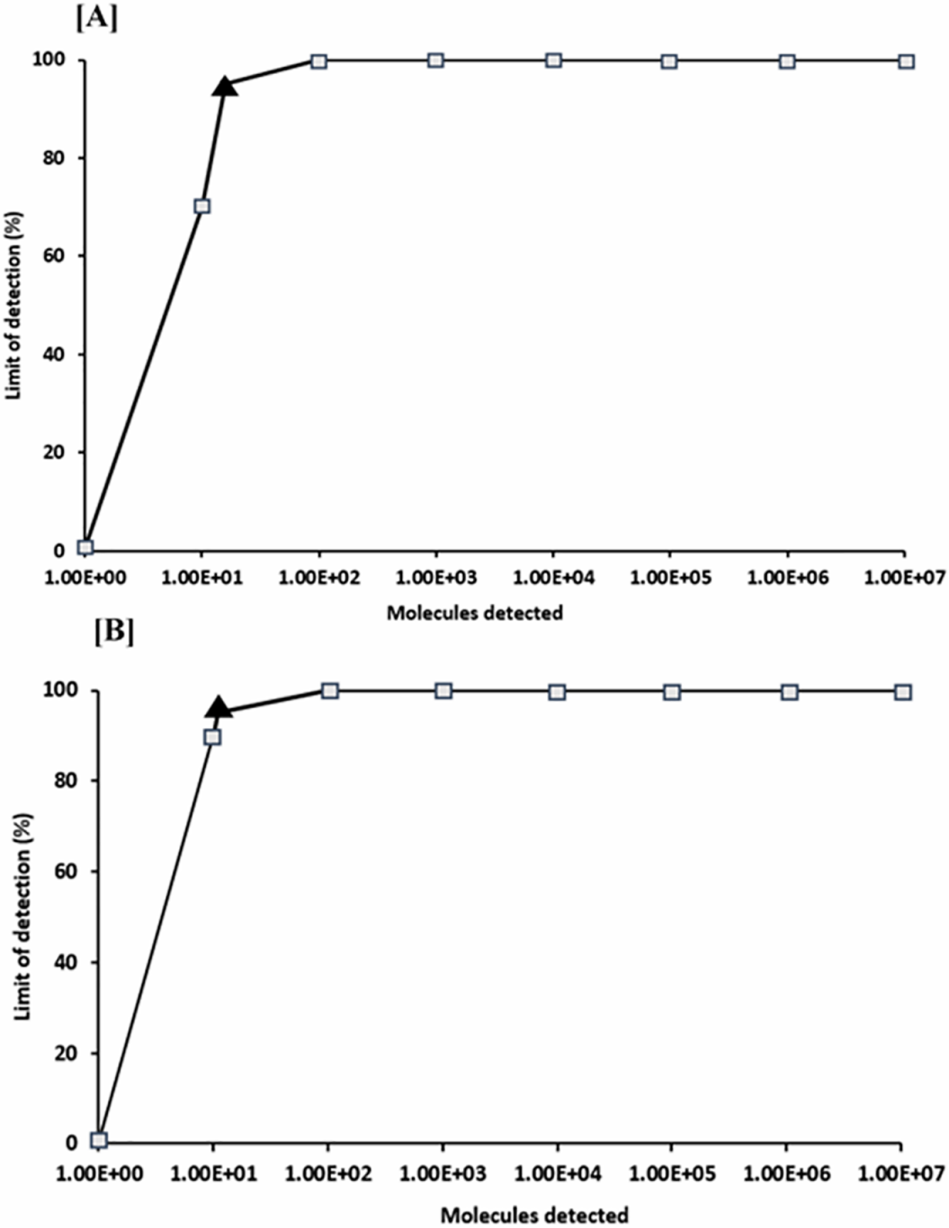
Probit regression analysis of data set of 10 runs of *Fno*-RPA and qPCR using Minitab^®^. The black triangle indicates limits of detection at 95% probability which were 15 and 11 molecules detected of *Fno*-plasmid standard DNA in RPA (A) and qPCR (B) respectively.

In the current study, we developed an isothermal RPA assay for rapid and specific detection of *Fno*. The analytical sensitivity of the developed RPA in our study was highly comparable to the published qPCR [22] which had a reported sensitivity of 10 DNA molecules of *Fno* gDNA [22]. We used a quantitative *Fno-*plasmid DNA dilutions range from 10^7^ to 10^1^ DNA molecules/**μ**L (Figs 2 and 3) in 10 independent runs for probit analysis which calculated detection limits of 15 and 11 DNA molecules for RPA (Fig 4A) and qPCR (Fig 4B), respectively. However, there was a contrast in the time required to reach the limits of detection in both assays, where RPA (Fig 2A) could achieve that in 6 min (6.2±0.6 min), needing only ~ 2.7–3 min to determine *Fno* in clinical samples, while qPCR (Fig 2B) required 90 min (Ct 35.2±0.6) to achieve its detection limit. The short time of detection in RPA makes it an attractive tool for an on-site detection and monitoring strategy for francisellosis especially on large farms. Also, the quick turnaround of RPA would likewise be of advantage in a standard research facility set-up empowering high-throughput testing.

The *Fno-*RPA developed in our study targeting the unique FSC771 hypothetical protein gene presents in *Fno*, but not in the closely related *Fnn* [22] showed high specificity, and only detected gDNA of *Fno* isolates, while all gDNA from other tested bacterial panel were negative (Table 3 and S2 Fig in supporting files).

**Table 3:** Analytical specificity of *Fno* RPA.

Bacterial species	Strain	Detection by RPA
*F*. *noatunensis* subsp. *orientalis*	STIR-GUS F2f7	+
*F*. *noatunensis* subsp. *orientalis*	NVI-PQ1104	+
*F*. *noatunensis* subsp. *orientalis*	DSMZ21254^**T**^	+
*F*. *noatunensis* subsp. *orientalis*	NVI-9449	+
*F*. *noatunensis* subsp. *orientalis*	AVU- Fran-Cos1	+
*F*. *noatunensis* subsp. *orientalis*	AVU- STIR-HON1	+
*F*. *noatunensis* subsp. *noatunensis*	NCIMB 14265 ^**T**^	-
*F*. *noatunensis* subsp. *noatunensis*	NVI-7601	-
*F*. *noatunensis* subsp. *noatunensis*	PQ1106	-
*F*. *philomiragia*	ATCC^®^ 25015 ^**T**^	-
*F*. *philomiragia*	ATCC^®^ 25017 ^**T**^	-
*F*. *philomiragia*	CCUG 12603 ^**T**^	-
*Aeromonas hydrophila*	ATCC^®^ 7966 ^**T**^	-
*Streptococcus agalactiae*	ATCC^®^ 51487 ^**T**^	-
*Streptococcus iniae*	ATCC^®^ 29178 ^**T**^	-
*Vibrio anguillarum*	ATCC^®^ 19264 ^**T**^	-
*Photobacterium damselae*	ATCC^®^ 51736 ^**T**^	-
*Escherichia coli*	ATCC^®^ 11775 ^**T**^	-
*Yersinia ruckeri*	ATCC^®^ 29473 ^**T**^	-
*Pseudomonas species*	Clinical isolate	-

^(T)^ Type strains

(+) Positive, (-) Negative.

We included three recently published alignments of *Fp* sequences [64] belonging to isolates recovered from water and human patients (accession no. CP009444; CP009442; CP009440) containing similar sequence of the FSC771 hypothetical protein gene in our analysis. In a previous cross detection experiment, the published qPCR did not pick up any *Fp* strains tested [22]. Sequences alignment now confirms that, this is due to 3 mismatches in the *Fp* target region for the qPCR probe.

Additionally, the cold-water fish pathogen *Francisella noatunensis* subsp. *noatunensis* used in the current cross-detection study, was found to be genetically, biochemically and morphologically distinct sub species from *Fno* [71]. Alignments of the available genome sequences [63–66] showed that *Fnn* was lacking the FSC771 hypothetical protein gene sequence. This finding is now confirmed as neither the developed RPA assay (Table 3) nor the previously published qPCR [22] could detect it. By using the principles of the ARMS concept [72] and including a mismatch at position 3 from the 3’-end of the RPA forward primer (Fig 1), detection of *Fp* was avoided as confirmed by our cross-detection assays (Table 3). Thus, even in the case of environmental contamination, false positive results due to pick up of *Fp* are excluded.

Testing the clinical samples by bacteriological tests, conventional PCR, qPCR and RPA showed a higher performance of RPA (Table 4). *Fno* was successfully recovered using CHAH from 7/10, 3/10 and 14/40 spleen samples from first and second UK and Thai tilapia, respectively. Also, *Fno-*positive tissues were detected by PCR (38/78 spleens, 33/78 head kidneys) (S3 Fig in supporting files), qPCR (46/78 spleens, 42/78 head kidneys) and RPA (47/78, 47/78). Interestingly, all water samples tested positive by RPA, while they were negative by PCR and qPCR (Table 4). The clinical sensitivity and specificity of the developed RPA assay were 100% and 84.93%, respectively (Table 5).

**Table 4:** Screening of tilapia tissues and water samples for *Fno*.

Sample ID.	Sample type	*Fno* isolation by bacteriology	Conventional PCR	qPCR Cycles threshold (Ct)		RPA threshold time (Tt) in minutes	
Fish 1	Spleen	-	+	30.06	+	3.7	+
	Head Kidney		+	36.97	+	5.7	+
Fish 2	Spleen	-	-	-	-	-	-
	Head Kidney		-	-	-	5.7	+
Fish 3	Spleen	+	+	30.8	+	5.7	+
	Head Kidney		+	25.1	+	3.3	+
Fish 4	Spleen	+	+	29.44	+	5.7	+
	Head Kidney		+	32.3	+	3.7	+
Fish 5	Spleen	+	+	27.45	+	3.3	+
	Head Kidney		+ (w)	31.07	+	5.7	+
Fish 6	Spleen	+	+	-	-	5.7	+
	Head Kidney		+	27.68	+	3.7	+
Fish 7	Spleen	+	+	32.38	+	5.7	+
	Head Kidney		-	-	-	5.7	+
Fish 8	Spleen	-	-	-	-	-	-
	Head Kidney		-	34.86	+	5.7	+
Fish 9	Spleen	+	+	38.82	+	5.7	+
	Head Kidney		+	30.16	+	5.7	+
Fish 10	Spleen	+	+	28.1	+	3.3	+
	Head Kidney		-	-	-	5.7	+
Fish 11	Spleen	-	-	-	-	-	-
	Head Kidney		-	-	-	6.0	+
Fish 12	Spleen	-	+	30.83	+	3.7	+
	Head Kidney		-	-	-	-	-
Fish 13	Spleen	-	-	-	-	-	-
	Head Kidney		-	-	-	-	-
Fish 14	Spleen		+	23.42	+	3.0	+
	Head Kidney		-	-	-	-	-
Fish 15	Spleen	+	+	27.98	+	2.7	+
	Head Kidney		+	25.14	+	3.3	+
Fish 16	Spleen	-	-	32.53	+	5.7	+
	Head Kidney		-	-	-	5.7	+
Fish 17	Spleen	+	+	28.19	+	3.0	+
	Head Kidney		-	-	-	-	-
Fish 18	Spleen	+	+	23.66	+	3.0	+
	Head Kidney		+ (w)	35.21	+	5.7	+
Fish 19	Spleen	-	-	-	-	-	-
	Head Kidney		-	-	-	-	-
Fish 20	Spleen	-	-	-	-	-	-
	Head Kidney		-	-	-	-	-
Fish 21	Spleen	+	+	23.94	+	5.7	+
	Head Kidney		+	21.24	+	4	+
Fish 22	Spleen	-	-	23.49	+	5.7	+
	Head Kidney		-	23.30	+	3.7	+
Fish 23	Spleen	+	+	23.57	+	5.7	+
	Head Kidney		+	20.51	+	3.3	+
Fish 24	Spleen	-	+	21.88	+	5.7	+
	Head Kidney		+ (w)	23.67	+	5.7	+
Fish 25	Spleen	+	+	22.19	+	5.7	+
	Head Kidney		+	22.34	+	3.7	+
Fish 26	Spleen	+	+	21.67	+	5.7	+
	Head Kidney		+	21.28	+	3.3	+
Fish 27	Spleen	-	+	22.91	+	5.7	+
	Head Kidney		+	19.49	+	5.7	+
Fish 28	Spleen	-	+	18.04	+	3.3	+
	Head Kidney		+	21.01	+	3.7	+
Fish 29	Spleen	-	-	19.99	+	5.7	+
	Head Kidney		-	22.33	+	3.7	+
Fish 30	Spleen	+	+	23.71	+	5.7	+
	Head Kidney		+	25.76	+	5.7	+
Fish 31	Spleen	+	+	21.81	+	3.7	+
	Head Kidney		+ (w)	30.94	+	5.7	+
Fish 32	Spleen	-	+	22.04	+	5.7	+
	Head Kidney		+ (w)	23.70	+	3.7	+
Fish 33	Spleen	+	+	24.41	+	3.7	+
	Head Kidney		+	21.85	+	3	+
Fish 34	Spleen	-	-	22.09	+	3.7	+
	Head Kidney		-	25.93	+	3.7	+
Fish 35	Spleen	+	+	24.66	+	3.7	+
	Head Kidney		+	20.32	+	3	+
Fish 36	Spleen	-	+	20.84	+	4	+
	Head Kidney		+	20.56	+	3	+
Fish 37	Spleen	-	-	21.78	+	3	+
	Head Kidney		-	26.36	+	5.7	+
Fish 38	Spleen	-	-	18.15	+	3	+
	Head Kidney		-	21.60	+	3.3	+
Fish 39	Spleen	+	+	24.65	+	5.7	+
	Head Kidney		+	22.90	+	4.7	+
Fish 40	Spleen	+	+	24	+	5.3	+
	Head Kidney		+	20.72	+	3.3	+
Fish 41	Spleen	+	+	20.81	+	3.3	+
	Head Kidney		+	21.14	+	3.7	+
Fish 42	Spleen	-	+	24.74	+	5.7	+
	Head Kidney		+	20.58	+	3	+
Fish 43	Spleen	-	-	29.67	+	5.7	+
	Head Kidney		-	22.37	+	3.3	+
Fish 44	Spleen	+	+	23.05	+	3.7	+
	Head Kidney		+	35.20	+	6	+
Fish 45	Spleen	-	+	29.06	+	5.7	+
	Head Kidney		+	21.52	+	3.3	+
Fish 46	Spleen	-	+	23.97	+	3.7	+
	Head Kidney		+	23.32	+	3.3	+
Fish 47	Spleen	+	+	20.32	+	3	+
	Head Kidney		+	22.73	+	3.3	+
Fish 48	Spleen	-	-	26.20	+	5.7	+
	Head Kidney		-	24.67	+	3.7	+
Fish 49	Spleen	-	+	22.54	+	3.7	+
	Head Kidney		+	21.96	+	3.3	+
Fish 50	Spleen	-	+	20.1	+	3	+
	Head Kidney		+	21.29	+	3	+
Fish 51	Spleen	-	+	21.59	+	3.3	+
	Head Kidney		+	18.75	+	3	+
Fish 52	Spleen	+	+	28.09	+	5.7	+
	Head Kidney		+	23.85	+	3.7	+
Fish 53	Spleen	-	-	-	-	-	-
	Head Kidney		-	-	-	-	-
Fish 54	Spleen	-	-	-	-	-	-
	Head Kidney		-	-	-	-	-
Fish 55	Spleen	-	-	34.53	+	5.7	+
	Head Kidney		-	33.9	+	5.7	+
Fish 56	Spleen	-	-	-	-	-	-
	Head Kidney		-	-	-	-	-
Fish 57	Spleen	-	-	-	-	-	-
	Head Kidney		-	-	-	-	-
Fish 58	Spleen	-	-	-	-	-	-
	Head Kidney		-	-	-	-	-
Fish 59	Spleen	-	-	-	-	-	-
	Head Kidney		-	-	-	-	-
Fish 60	Spleen	-	-	-	-	-	-
	Head Kidney		-	-	-	-	-
Fish 61	Spleen	N/A	-	-	-	-	-
	Head Kidney		-	-	-	-	-
Fish 62	Spleen	N/A	-	-	-	-	-
	Head Kidney		-	-	-	-	-
Fish 63	Spleen	N/A	-	-	-	-	-
	Head Kidney		-	-	-	-	-
Fish 64	Spleen	N/A	-	-	-	-	-
	Head Kidney		-	-	-	-	-
Fish 65	Spleen	N/A	-	-	-	-	-
	Head Kidney		-	-	-	-	-
Fish 66	Spleen	N/A	-	-	-	-	-
	Head Kidney		-	-	-	-	-
Fish 67	Spleen	N/A	-	-	-	-	-
	Head Kidney		-	-	-	-	-
Fish 68	Spleen	N/A	-	-	-	-	-
	Head Kidney		-	-	-	-	-
Fish 69	Spleen	N/A	-	-	-	-	-
	Head Kidney		-	-	-	-	-
Fish 70	Spleen	N/A	-	-	-	-	-
	Head Kidney		-	-	-	-	-
Fish 71	Spleen	N/A	-	-	-	-	-
	Head Kidney		-	-	-	-	-
Fish 72	Spleen	N/A	-	-	-	-	-
	Head Kidney		-	-	-	-	-
Fish 73	Spleen	N/A	-	-	-	-	-
	Head Kidney		-	-	-	-	-
Fish 74	Spleen	N/A	-	-	-	-	-
	Head Kidney		-	-	-	-	-
Fish 75	Spleen	N/A	-	-	-	-	-
	Head Kidney		-	-	-	-	-
Fish 76	Spleen	N/A	-	-	-	-	-
	Head Kidney		-	-	-	-	-
Fish 77	Spleen	N/A	-	-	-	-	-
	Head Kidney		-	-	-	-	-
Fish 78	Spleen	N/A	-	-	-	-	-
	Head Kidney		-	-	-	-	-
Water Samples	UV filter	N/A	-	-	-	5.7	+
	Bio-filter 1	N/A	-	-	-	5.7	+
	Bio-filter 2	N/A	-	-	-	3.7	+
	Tank 1	N/A	-	-	-	6	+
	Tank 2	N/A	-	-	-	5.7	+

(+) Positive, (-) Negative, (w) Weak positive/negative, (N/A) Not done

**Table 5:** The diagnostic performance of the *Fno* RPA using field samples.

Real-time qPCR					Performance characteristics (%)			
Method	True positive	True Negative	False Positive	False negative	Sensitivity % (95% CI)	Specificity % (95% CI)	PPV% (95% CI)	NPV% (95% CI)
RPA	88	62	11	0	100% (95.89 to 100)	84.89% (74.64 to 92.23)	88.89% (82.27 to 93.24)	100% (100)

(PPV: Positive predictive value, NPV: Negative predictive value)

The robustness of the RPA to crude clinical specimens is often featured as a favourable benefit. The developed RPA in our study was found to be more robust than qPCR in detection of *Fno* when clinical/field samples were used. The RPA scored more positive results than qPCR which failed to detect *Fno* in 11 crude DNA samples (1 spleen, 5 head kidney and 5 water samples) and only showed positive reactions after their dilution (S1 Table in supporting files). This is due to the fact that, RPA is more tolerant to common PCR reaction inhibitors [36]. This advantage was highlighted in various studies, which showed that RPA can work in presence of agents with inhibitory effect on PCR including 15–25% of milk, 50 g/L haemoglobin, 4% V/V ethanol and 0.5 U of heparin [36]. Also, detection of 10^3^ molecules of gDNA of *S*. *agalactiae* in presence of up to 5 **μ**L of stool sample and 5x10^6^ molecules of Crimean-Congo haemorrhagic fever viral RNA in preparations contained 1:10 dilutions of crude human serum, urine and tick pool homogenate was previously reported [75, 76]. This finding highlights the robustness of the developed *Fno* RPA for detection of nucleic acids in different crude biological samples if the appropriate extraction protocol is carried out.

Moreover, other isothermal assays were adopted for diagnosis of francisellosis in tilapia including LAMP that was successfully used for detection of *Fno* with LOD at 1 fg [33]. However, LAMP has many drawbacks in comparison with RPA, as it depends on turbidity index measurement with a Loopamp® Realtime Turbidimeter that weighs ~ 5 Kg, uses a complex-design of 6 oligonucleotides targeting four target sequences, requires a high temperature of 60°C, and has a longer reaction time (45 min) [33]. Thus, these findings ultimately favour the usage of RPA instead of LAMP for mobile isothermal detection of *Fno*.

The *Fno-*RPA was performed at a relatively low temperature with isothermal conditions (42°C) and real-time monitoring was performed using an ESE-Quant tube scanner which is less expensive than a mobile cycler. This tube scanner is convenient with a footprint of 17.5 x 19 cm and an approximate weight of 2 Kg including the attached laptop. Other readers are commercially available such as the Axxin TSO-ISO reader [73] or the Genie III [74]. These devices or others currently being developed such as hand-held detection devices can contribute to the development of mobile pond-side or point-of-care diagnosis of *Fno* in tilapia farms.

One of the main benefits of the RPA is the convenience of the assay, as the kit used (TwistAmp Exo kit) is commercially available in the form of dried pellets accompanied with the rehydration buffer and reagents required for the reaction mixture. The only step required is the addition of primers, probe and template DNA. Also, the detection is performed with an ESE-Quant tube scanner that is very compact. Nevertheless, a major constraint of using the RPA in field is ability to extract good quality nucleic acids to perform the test. However, there are many commercially available DNA extraction methods at the moment which are simple, cheap and suitable for field application including magnetic bead-based technology, heated NaOH method [77] and mobile extraction devices like QuickGene-Mini80 (Autogen^®^, USA) [78, 79]. Using any of these methods will make a considerable decrease in the cost of the RPA assay and provide more flexibility to its use in the field conditions and in the poor-setting diagnostic labs.

Recently, RPA assays were used in combination with other tools including lateral flow dip sticks (LFD) [77, 80–82], enzyme-linked immunosorbent assays [83], aptamer-based bio-barcodes (ABC) [84], and hybridization in microarray format [85]. These tools enhanced the performance of the RPA assay and elucidated its significance as a versatile next-generation molecular diagnostic test. Overall, the *Fno-*RPA developed in the current study can be considered a potential user-friendly method for the simple, accurate and rapid detection of *Fno* that can be applied for field screening of tilapia for francisellosis.

## Conclusions

A novel real-time *Fno*-RPA was developed for the rapid and accurate detection of *Fno* that showed high analytical sensitivity and specificity with robust performance when applied in clinical samples. The sensitivity, specificity and reproducibility were highly comparable to the published qPCR with better tolerance to amplification inhibitors. Using the RPA assay with a mobile tube scanner and a fast and affordable DNA extraction protocol could be used as a powerful “pond-side test” to be applied on fish farms in poor settings infrastructure for the detection of *Fno*. Future studies need to be conducted to test different DNA extraction methods for further improvement of the assay application.

## Supporting information

S1 Fig *Fno* standard plasmid map. (TIF)

S2 Fig Analytical specificity of the *Fno* RPA. Positive amplification only with *Fno* isolates [A], while negative results were obtained with *Fnn*, *Fp* and non-*Francisella* isolates [B and C]. A1 (violet line), B1 (Blue line) and C1 (blue line): Positive control, A2: Negative control (dark green line), A3: A8: *Fno* isolates (A3: *Fno* UK isolate (black line), A4: *Fno* Costa Rican isolate (red line), A5: *Fno* Japanese isolate (green line), A6: *Fno* Austrian isolate (orange line), A7: *Fno* Mexican isolate (pink line), A8: *Fno* Central American isolate (brown line), B2: B7: *Fnn* isolates (B2: *Fnn* Norwegian isolate (black line), B3: *Fnn* Irish isolate (red line), B4: *Fnn* Swedish isolate (green line), *Fp* isolates (B5: *Fp* from muskrat (orange line), B6: *Fp* from water (pink line), B7: *Fp* human (brown line) and B8: *A*. *hydrophila* (dark green line). C2: *S*. *agalactiae* (black line), C3: *S*. *iniae* (red line), C4: *V*. *anguilarum* (green line), C5: *P*. *damselae* (orange line), C6: *E*.*coli* (pink line), C7: *Y*. *ruckeri* (brown line), C8: *Pseudomonas spp*. (dark green line).
(TIF)

S3 Fig PCR results for 156 tissue samples from tilapia and 5 water samples collected from UK and Thailand farms after electrophoresis on 1% agarose gel. M: 100Pb DNA marker, S: spleen, K: Head kidney, UV: ultraviolet filter, B1: Bio-filter tank 1, B2: Bio-filter tank 2, T1: Fish tank 1, T2: Fish tank2, PC: Positive control (*Fno* gDNA), NTC: Negative control (Milli-Q water).
(TIF)

S1 Table Results of testing diluted crude DNA preparations from fish tissues and water samples by qPCR. (DOCX)
