# Supplementary figures and images for: Development of a recombinase polymerase amplification assay for rapid detection of Francisella noatunensis subsp. orientalis
Source: PLoS One. 2018 Feb 14;13(2):e0192979. doi: 10.1371/journal.pone.0192979 (PMC5812721; doi:10.1371/journal.pone.0192979)

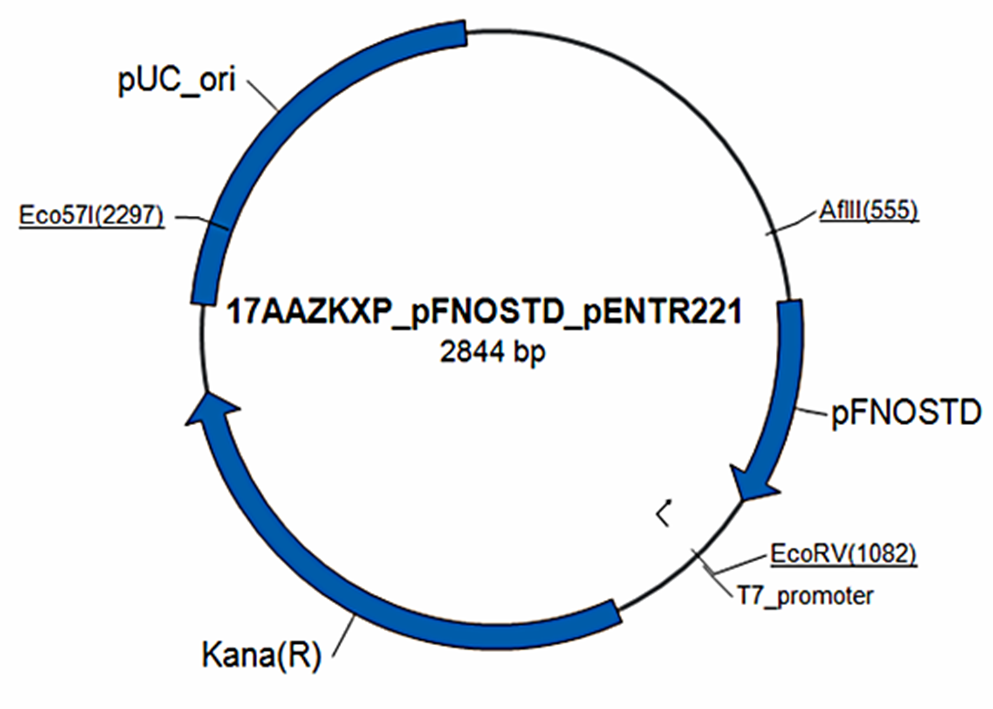

Supplement: S1 Fig — (TIF) [file pone.0192979.s001.tif]

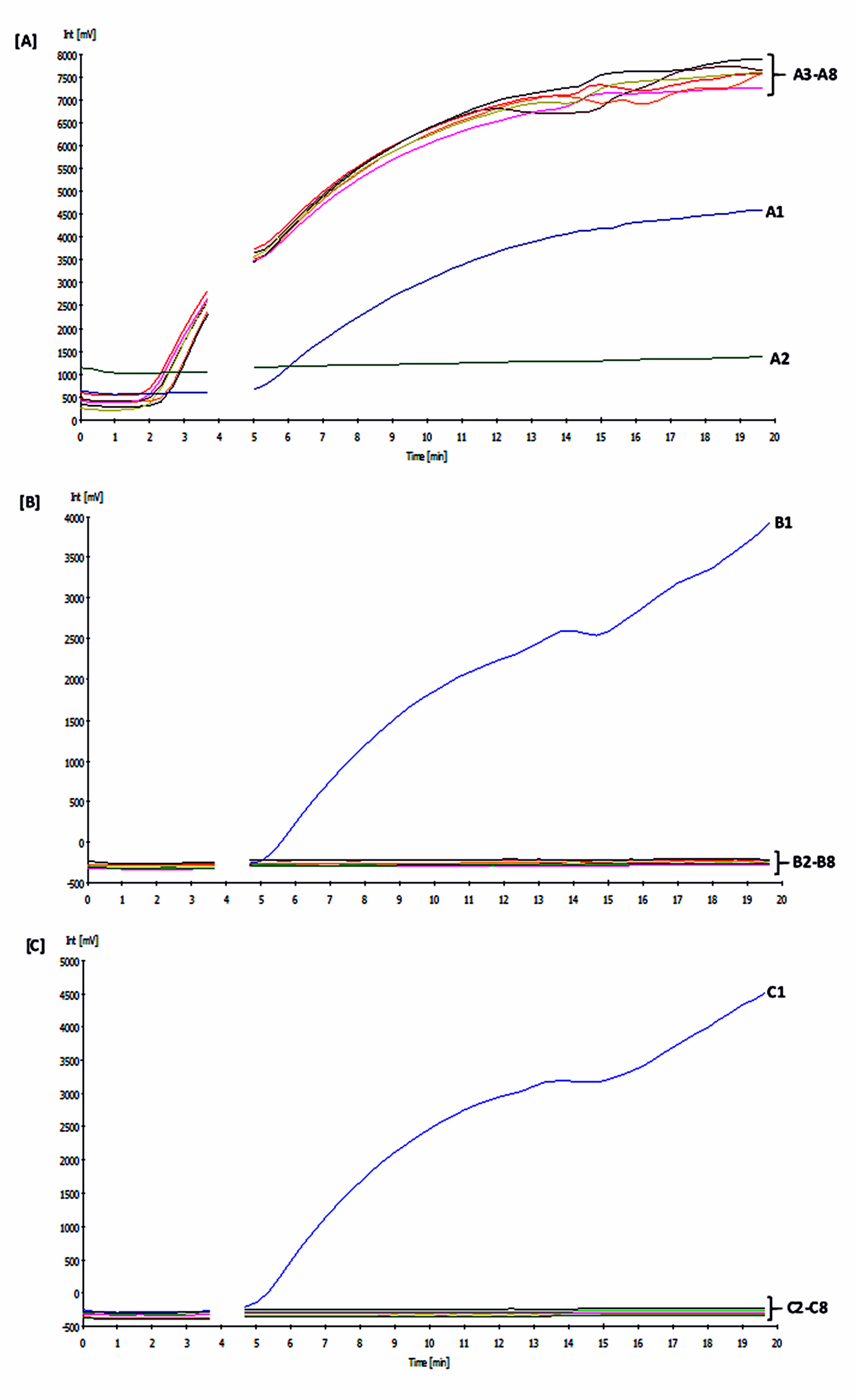

Supplement: S2 Fig — Positive amplification only with Fno isolates [A], while negative results were obtained with Fnn, Fp and non-Francisella isolates [B and C]. A1 (violet line), B1 (Blue line) and C1 (blue line): Positive control, A2: Negative control (dark green line), A3: A8: Fno isolates (A3: Fno UK isolate (black line), A4: Fno Costa Rican isolate (red line), A5: Fno Japanese isolate (green line), A6: Fno Austrian isolate (orange line), A7: Fno Mexican isolate (pink line), A8: Fno Central American isolate (brown line), B2: B7: Fnn isolates (B2: Fnn Norwegian isolate (black line), B3: Fnn Irish isolate (red line), B4: Fnn Swedish isolate (green line), Fp isolates (B5: Fp from muskrat (orange line), B6: Fp from water (pink line), B7: Fp human (brown line) and B8: A. hydrophila (dark green line). C2: S. agalactiae (black line), C3: S. iniae (red line), C4: V. anguilarum (green line), C5: P. damselae (orange line), C6: E.coli (pink line), C7: Y. ruckeri (brown line), C8: Pseudomonas spp. (dark green line). (TIF) [file pone.0192979.s002.tif]

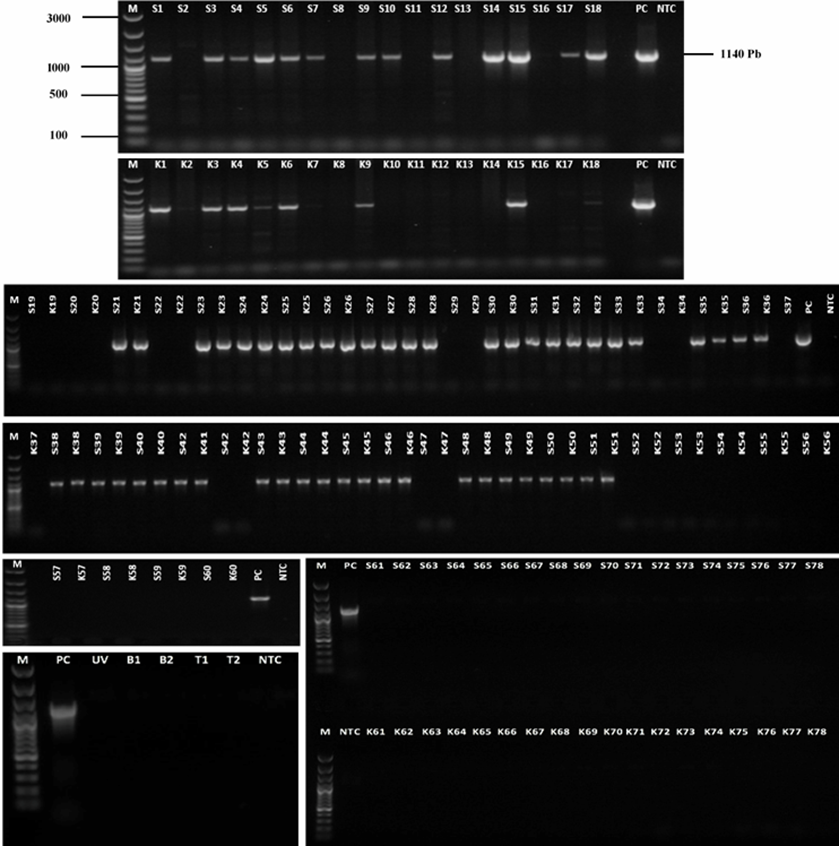

Supplement: S3 Fig — M: 100Pb DNA marker, S: spleen, K: Head kidney, UV: ultraviolet filter, B1: Bio-filter tank 1, B2: Bio-filter tank 2, T1: Fish tank 1, T2: Fish tank2, PC: Positive control (Fno gDNA), NTC: Negative control (Milli-Q water). (TIF) [file pone.0192979.s003.tif]
