# Supplementary material for: Development of a recombinase polymerase amplification assay for rapid detection of Francisella noatunensis subsp. orientalis
Source: PLoS One. 2018 Feb 14;13(2):e0192979. doi: 10.1371/journal.pone.0192979 (PMC5812721; doi:10.1371/journal.pone.0192979)
Supplement: S1 Table — (DOCX) [file pone.0192979.s004.docx]

**S1 Table. Results of testing diluted crude DNA preparations from fish tissues and water samples by qPCR.** *

**M**

| Sample | Crude DNA | | 1:10 dilution | | 1:100 dilution | |
| --- | --- | --- | --- | --- | --- | --- |
|  | Ct value | Interpretation | Ct value | Interpretation | Ct Value | Interpretation |
| Spleen 6 | N | - | 26.95 | + | 30.04 | + |
| Head Kidney 2 | N | - | 29.50 | + | 32.88 | + |
| Head Kidney 7 | N | - | 30.20 | + | 34.30 | + |
| Head Kidney 10 | N | - | 31.14 | + | 33.54 | + |
| Head Kidney 11 | N | - | - | - | - | - |
| Head Kidney 16 | N | - | 34.04 | + | - | - |
| UV filter water | N | - | 34.88 | + | 37.02 | + |
| Bio-filter 1 water | N | - | - | - | - | - |
| Bio-filter 2 water | N | - | 34.99 | + | 36.72 | + |
| Tank 1 water | N | - | 36.80 | + | 39.9 | + |
| Tank 2 water | N | - | - | - | - | - |

(+) Positive, (-) Negative, (Ct) cycle threshold.

* All results are mean out of duplicate reactions.
